# Characterization of the pVHL Interactome in Human Testis Using High-Throughput Library Screening

**DOI:** 10.3390/cancers14041009

**Published:** 2022-02-17

**Authors:** Antonella Falconieri, Giovanni Minervini, Federica Quaglia, Geppo Sartori, Silvio C. E. Tosatto

**Affiliations:** 1 Department of Biomedical Sciences, University of Padova, 35121 Padova, Italy; antonella.falconieri@unipd.it (A.F.); giovanni.minervini@unipd.it (G.M.); geppo.sartori@unipd.it (G.S.); 2 Institute of Biomembranes, Bioenergetics and Molecular Biotechnologies, National Research Council (CNR-IBIOM), 70126 Bari, Italy; f.quaglia@ibiom.cnr.it

**Keywords:** VHL, von Hippel–Lindau syndrome, ubiquitination, hypoxia, testis, yeast two hybrid (Y2H)

## Abstract

**Simple Summary:**

The von Hippel–Lindau (pVHL) tumor suppressor is a protein that regulates the normal cell adaptation to low oxygen concentrations. When its function is altered by inherited or acquired mutation pVHL becomes causative of a familiar predisposition to develop different types of cancers. Besides this role, pVHL is also thought to have other relevant cell functions, and studies in mice demonstrated that this protein is crucial for correct testis development and sperm maturation. By scanning the testis-specific library, we identified 55 novel proteins that interact with the human pVHL, with many of them directly participating in metabolic pathways frequently altered in cancer. Furthermore, our results suggest that pVHL may be also important for correct gonad function in men.

**Abstract:**

Functional impairment of the von Hippel–Lindau tumor suppressor (pVHL) is causative of a familiar increased risk of developing cancer. As an E3 substrate recognition particle, pVHL marks the hypoxia inducible factor 1α (HIF-1α) for degradation in normoxic conditions, thus acting as a key regulator of both acute and chronic cell adaptation to hypoxia. The male mice model carrying VHL gene conditional knockout presents significant abnormalities in testis development paired with defects in spermatogenesis and infertility, indicating that pVHL exerts testis-specific roles. Here we aimed to explore whether pVHL could have a similar role in humans by performing a testis-tissue library screening complemented with in-depth bioinformatics analysis. We identified 55 novel pVHL binding proteins directly involved in spermatogenesis, cell differentiation and reproductive metabolism. In addition, computational investigation of these new interactors identified multiple pVHL-specific binding motifs and demonstrated that somatic mutations described in human cancers reside in these binding regions. Collectively, these findings suggest that, in addition to its role in cancer formation, pVHL may also be pivotal in normal gonadal development in humans.

## 1. Introduction

The von Hippel–Lindau (VHL) disease (OMIM number 193300) is a rare autosomal dominantly inherited genetic disorder [1] with an incidence of 1:36,000 live births and high penetrance of >90% by age 65 with a mean age at tumor diagnosis of 26 [2]. The VHL disease is a multiple-neoplasia disorder associated with the development of highly vascular tumors such as retinal and CNS hemangioblastoma (HBs), clear cell renal cell carcinoma (RCC), pheochromocytoma (PCC) and paraganglioma (PGL), pancreatic cystadenomas, endolymphatic sac tumors (ELSTs), epididymal cysts and broad ligament cystadenoma [3]. Clinical VHL manifestations are variable, with different phenotypes associated with the same mutation in different families or even in the same family [3]. The disease arises from pathogenic inactivation of the homonymous VHL gene located on the short arm of chromosome 3 (3p25-26) [4]. In humans, VHL encodes two protein products, a 30 kDa full-length protein named pVHL30 and a shorter isoform referred to as pVHL19 that is generated by an alternative translation initiation site at methionine 54 [5]. A third isoform, known as pVHL172, arises from an alternative spliced mRNA in which the exon E2 is excluded [6]. Full-length pVHL30 presents three distinct domains: An intrinsically disordered N-terminal tail (aa1–53), a central full β-domain (aa54 to 157) and a C-terminal α-domain (aa158 to 213) [7,8]. In normoxic conditions, pVHL controls degradation of the hypoxia inducible factor 1α (HIF-1α) [7] acting as the substrate recognition particle of a ubiquitin ligase (E3) multiprotein complex, formed with Elongin B, Elongin C, Cullin2 and Rbx1 [9,10]. Interaction of pVHL with HIF-1α is highly specific and triggered by HIF-1α proline hydroxylation mediated by the prolyl-4 hydroxylase domain containing enzymes (PHD1, -2 and -3), while it is inhibited under hypoxic conditions [7,11]. Apart from its role in degrading HIF-1α, multiple alternative functions have been linked to pVHL such as to promote microtubules stabilization, regulate kinases activity, participate in the formation of the extracellular matrix, as well as to regulate cell senescence and apoptosis [12,13,14,15,16]. The pVHL is frequently described as a molecular hub that mediates the interactions with more than 500 binding partners involved in multiple cell pathways [17,18]. The biological meaning of most of these interactions remains, however, poorly understood. Male mice carrying VHL conditional knockout present abnormalities in testis development, reduced sperm count, impaired spermatogenesis and infertility, besides altered vascularization in multiple organs which is a conventional marker of nonfunctional pVHL [19]; 25%–60% of VHL male patients develop epididymal papillary cystadenomas [20,21], an otherwise rare form of neoplasm affecting male reproductive organs [22]. In contrast with the VHL murine model, no infertility or defect of spermatogenesis are reported in humans except for mechanical obstruction of seminal ducts due to epididymal cysts. On the other hand, pVHL is required for the correct cilia formation [23], while it is well understood that multiple male infertility syndromes arise from congenital defects in ciliogenesis [24]. Similarly, defects in ciliogenesis are also reported in cancer and linked to deregulation of the cellular response to signals from multiple pathways [25,26].

Here, we describe the interactome around the human pVHL obtained through testis-tissue library screening by yeast mating. We identified 55 novel testis-specific pVHL30 interactors, with multiple proteins directly involved in spermatogenesis and reproductive metabolism, and which also play a role in cancer formation. 

## 2. Materials and Methods

### 2.1. Yeast Two-Hybrid (Y2H) Assay and Bait Vector Construction

The yeast two-hybrid (Y2H) assay is a simple yet powerful method to detect binary protein–protein interactions (PPis) by exploiting the Gal4 transcription factor modularity. In this system the two proteins of interest, referred to as bait, are fused to the Gal4-DNA binding domain and Gal4-activating domain, respectively. If the bait and prey proteins interact, the Gal4 transcription factor is reconstituted and activates the transcription of a reporter gene, leading to yeast survival on selective conditions. cDNA encoding pVHL30 was cloned into the pGBKT7 vector using the in vitro In-Fusion^®^ method. The full-length cDNA encoding of the human pVHL30 was transferred from pCDNA3.1 purchased by GenScript, Piscataway, NJ, USA (GenEZ plasmid OHu23297) to a pGBKT7 empty vector (Takara Clontech, Dalian, China). The cDNA was amplified by PCR using specific primers carrying 15 base long 5’ ends corresponding to the regions around the EcoR1 site in the MCS of the bait vector. Then, the PCR was recombined with a pGBKT7 linearized vector (by EcoR1 digestion) using the In-Fusion HD cloning Kit (Takara Clontech) according to the manufacturer’s protocols. The resulting recombinant plasmid pGBKT7-pVHL30 was sequenced by the Sanger method and checked for protein expression by western blot.

### 2.2. Library Screening by Yeast Mating

The yeast library screening was performed using the Mate & Plate™ Library—Human Testis (Takara Clontech, Cat. n° 630470). According to this, the library is expressed in the Y187 yeast strain (MATa); conversely, the bait protein was expressed in Y2HGold, a MATα yeast strain. A fresh colony of a bait strain (Y2HGold (pGBKT7-pVHL30)) was inoculated in SD-Trp with shaking at 140 rpm o/n at 30 °C. When the OD600 reached 0.8, the culture was centrifugated and the pellet re-suspended in 4 ml of SD-Trp. In a sterile 3-L flask, 4 ml of bait strain was combined with 1 ml aliquot of the testis library and 45 ml of YPDA 2 x liquid medium at 30 °C with slow shaking (around 30–50 rpm). After 30 h cells were centrifugated and the pellet re-suspended in 10 ml of 0.5 × YPDA liquid medium and plated on 150 mm SD-Leu-Trp + X-α-Gal + AbA (200 μL per plate). Plates were incubated at 30 °C for 5 days monitoring colony growth twice a day. All positive clones were patched out on more stringency medium QDO + X-α-Gal + AbA in order to avoid false positives. The yeast host strains used were purchased from Takara Clontech: Y2HGold (genotype: MATa, trp-901, leu2-3, 112, ura3-52, his3-200, gal4Δ, gal80Δ, LYS2::GAL2UAS-Gal1TATA-HIS3,GAL2UAS-Gal12TATA-Ade2, URA::MEL1UAS-Mel1TATA AUR1-c MEL1) and Y187 (genotype: MATα, ura3-52, his3-200, ade2-101, trp1-901, leu2-3, 112, gal4Δ, met–, gal80Δ, URA3::GAL1UAS-GAL1TATA-lacZ).

### 2.3. Positive Clone Analysis

DNA was extracted from yeast colonies using the Zymoprep™-96Yeast Plasmid Miniprep (Zymo Research, Irvine, CA, USA Cat. D2005) and 2 µl of each DNA was used to transform TOP10 E. Coli (calcium chloride competent cells homemade) according to standard protocol. Five colonies of each transformation were analyzed. In this case, plasmidic DNAs were extracted using Zyppy TM-96 Plasmid Miniprep kit (Zymo Research Cat. D4041) and used as templates to amplify cDNA inserts with Gal4AD For and Gal4AD Rev primers. PCRs were performed according to Wondertaq protocol (Euroclone, Milano, Italy Cod. EME020001) and visualized on 0.8% agarose gel. All positive clones with only one PCR product were transformed in the Y190 yeast strain, provided by Euroscarf, Obersursel, Germany (genotype: MATa, gal4-542, gal80-538, his3, trp1-901, ade2-101, ura3-52, leu2-3, 112, URA3::GAL1-LacZ, Lys2::GAL1-HIS3cyhr). Each clone was co-transformed with a pGBKT7 empty vector (as auto-activation control) and with pGBKT7-pVHL30 (as interaction control). For each transformation plate, two colonies were patched out on a permissive medium (SD-Trp-Leu) and on selective ones (SD-Leu-Trp-His + 30 mM or 60 mM 3AT). Finally, using the Gal4AD For primer, all positive clones were sequenced by the Sanger method.

### 2.4. Positive Clone Identification and Bioinformatic Analysis

To identify all the positive clones, nucleotide sequences were translated with Expasy Translate to retrieve the corresponding amino acid sequences. Resulting amino acid sequences were analyzed with BLASTP [27] against the UniProtKB database [28] (default options). Multiple sequence alignment to identify shared banding regions was performed with Jalview [29]. From a starting library including 142 clones, a total of 61 pVHL30 interactors were identified. The final dataset includes 6 pVHL30 interactors already described in the literature, while the remaining 55 are newly identified. Data about molecular function and subcellular localization for each interactor were retrieved from UniProtKB and Gene Ontology (GO) [30]. The protein–protein interaction network around the novel pVHL30 interactors was generated with Cytoscape [31], using molecular data from STRING [32] and BioGRID [33]. The preliminary network was extended with a second shell of STRING interactors retrieved setting parameters as follows: ≤50 interactors, confidence ≥ 0.400. MCODEs [34] were used to identify clusters of highly interconnected proteins. Analysis of biochemical pathways, GO terms and disease data was performed with Enrichr [35] and ClueGo [36]. Conservation of the core interaction networks across different tissues was investigated with HumanBase [37].

## 3. Results

### 3.1. Yeast Two-Hybrid (Y2H) Library Screening in Testis Tissue

To investigate the pVHL30 interactome in testis tissue we performed a large-scale screening using the yeast *S. cerevisiae* as the organism model. The Y187 haploid (MATa) yeast strain expressing a human testis cDNA library (Takara Clontech) was mated to the Y2HGold haploid strain (MATα) expressing pVHL30 as the bait protein. To generate the bait, we cloned the cds of pVHL30 in the pGBKT7 vector as described in the Materials and Methods section. The vector encodes the pVHL30 protein fused to the Gal4-DNA binding domain as verified by western blot (Supplementary Figure S1).

**Figure 1:**
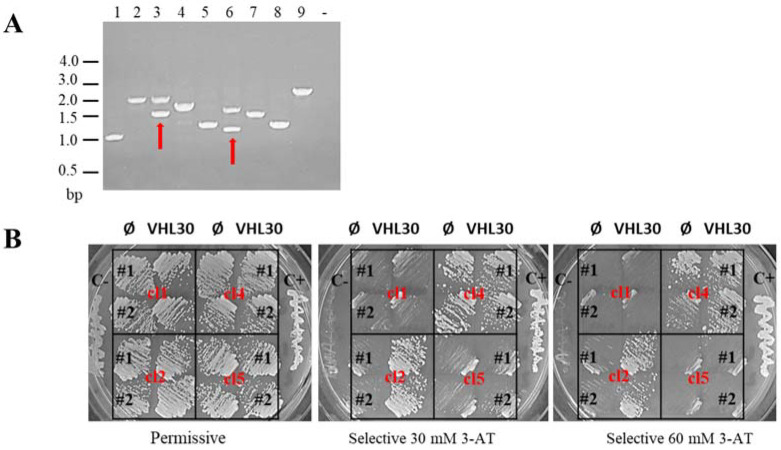
Analysis of Y2H library positive clones. (**A**) PCR fragment visualization on agarose gel. Each lane is marked with a number (n) corresponding to a library positive clone. The last column corresponds to the PCR negative control (-). Red arrows indicate clones with more than one insert, i.e., 3 and 6. (**B**) Yeast patches. All positive clones were co-transformed with pGBKT7 empty (Ø) or pGBKT7 VHL30 (VHL30) as indicated on the top row. Two independent colonies (#1, #2) of each transformation were tested on permissive (right) and selective (left) media. C+ and C- correspond to Y2H positive and negative control, respectively.

First, we checked for pVHL30 expression and its toxicity to exclude any alteration in yeast colonies’ growth-rate and their dimension. The growth of the Y2HGold strain expressing pGBKT7VHL30 and prey empty vector was analyzed to exclude unspecific autoactivation and confirm that the yeast growth detected on selective media was dependent upon prey interaction. Mated culture was plated on selective media and plates incubated for five days at 30 °C monitoring colony number during the time. To reduce false positives, each positive clone identified after the mating was patched out on a more selective medium (Figure 1). All these procedures allowed us to detect 607 positive clones. Total yeast DNA (i.e., genome, bait and prey plasmids) was extracted from each of 607 yeast clone and analyzed by PCR using two primers able to pair vector sequences upstream and downstream the multiple cloning sites of the pGADT7 plasmid. Yeast clones characterized by the presence of multiple recombinant plasmids carrying different inserts were excluded from the identification process. All the other yeast DNAs were transformed in *E. coli* in order to amplify and isolate DNAs of prey plasmids. Finally, to further validate the pVHL30 interactors in a different genetic background each prey plasmid DNA was co-transformed with pGBKT7, empty or expressing pVHL30, in a Y190 yeast strain and analyzed on selective media. 

At the end of all these steps we detected 260 positive clones corresponding to an equal number of nucleotide sequences determined by Sanger sequencing. 

### 3.2. Identification and Characterization of pVHL30 Interactors

Resulting cDNA sequences were translated with ExPASy Translate and the amino acid sequences were used to perform BLAST search in UniProt. This step retrieved matches for 142 sequences. Sequences corresponding to putative pVHL30 binding motifs were identified from the library screening and mapped on the corresponding full-length protein to define the specific fragments involved (Table 1). Considering the presence of multiple clones encoding for the same protein, a total of 61 different pVHL30 interactors were identified from the library dataset. In detail, six proteins are already known pVHL interactors, whereas the remaining 55 candidates correspond to novel pVHL30 binding partners (Table 1 and Supplementary Table S1). According to VHLdb [18] the six known interactors are EEF1A1, CCT5, CCT7, Elongin-C, SNRNP200 and UBE2D2. 

**Figure 2:**
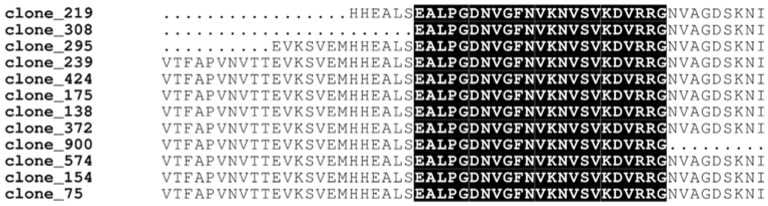
EEF1A1 multiple sequence alignment. Amino acid sequences corresponding to EEF1A1 (UniProt_ID P68104) derived from different clones. The region shared among all clones highlights the putative pVHL30 binding motifs.

We then wondered whether fragments corresponding to the same protein could be used to refine identification of pVHL binding motifs. In particular, we assumed that these fragments may include the same binding region which in turn corresponds to the minimal amino acid region responsible for the interaction. To this end, we extracted candidate interactors presenting more than two occurrences and aligned their sequences to identify shared regions—if any—among different clones. As an example of the analysis workflow, we report the alignment generated using fragments belonging from the Elongation factor 1-alpha 1 (EEF1A1), the most represented protein in our dataset (Figure 2**)**.

We identified 17 hits corresponding to 12 different amino acid sequences. These were aligned and compared with the EEF1A1 reference sequence retrieved by UniProt [28] (ID P68104). We found a fragment of 27 residues (aa297–324) shared among all clones (Figure 2), suggesting this region was the pVHL30 binding motif. Following this approach for each interactor, a total of 12 different promising pVHL30 binding fragments were identified (Supplementary Table S2). In our library screening we also identified multiple fragments from the same protein sharing no significant sequence identity, suggesting that each of them possesses unique features. These fragments were then investigated for secondary structure content, presence of functional domains, functional linear motifs and post-translational modification sites. Interestingly, by searching against the ELM database [38] we found the presence of a so-called “USP7-binding motif” in three proteins, i.e., NOP2/Sun RNA Methyltransferase Family Member 7 (NSUN7), Spermatogenesis Associated 22 (SPATA22) and Protein Disulfide Isomerase Family A Member 3 (PDIA3). The Ubiquitin Specific Peptidase 7 (USP7) is a deubiquitinating enzyme involved in p53 stabilization [39] presenting high affinity for MDM2, the main E3 ubiquitin-ligase involved in p53 degradation [40]. pVHL30 was recently proposed to bind MDM2 up-stream to the USP7 interacting motif [41]. Moreover, pVHL30 is also known to interact with both USP33 and USP20, two enzymes belonging to the USP protein family, whose USP7 is a further member [42]. Data from the literature indicate that pVHL30 mediates the ubiquitination and degradation of both USP33 and USP20, in turn down-regulating the pathways controlled by these two proteins [43]. These findings may suggest that pVHL30 plays a role in regulating MDM2/USP7 association.

### 3.3. Predicted Sub-Cellular Distribution of the New Identified pVHL30 Interactors

Based on the UniProt database [28], the new pVHL30 binding partners were found to preferentially localize into the nucleus (29 interactors), while seven are cytosolic proteins. The remaining identified interactors are almost equally distributed among endosome, membrane, chromosome and endoplasmic reticulum. Only one protein was found to participate in the lysosome, i.e., cathepsin D (CTSD), whereas no interactors residing in the Golgi were found. Interestingly, among the novel putative pVHL30 interactors, we also identified four mitochondrial proteins, i.e., ETFA, MRPS9, MTCO2 and PHB2, mainly involved in the regulation of mitochondrial respiration activity and electron transfer to the mitochondrial respiratory chain. The predominance of nuclear proteins is not surprising per se as the nucleus represents one of the subcellular compartments in which pVHL is normally found at physiological conditions. Furthermore, the pVHL sub-localization is thought to be regulated in a cell cycle-dependent manner that influences the protein shuttling between the nucleus and the cytoplasm [44]. The pVHL30 association with CTSD is, nevertheless, particularly interesting. A recent work demonstrated a lysosomal vulnerability in VHL-inactivated RCC cells, at least in vitro [45]. In other words, renal cancer cells possessing unfunctional pVHL seem unable to maintain their lysosome localization upon drug-induced stress. We propose that the association identified here between pVHL30 and CTSD may play a role in renal cancer progression. 

### 3.4. Prediction of pVHL30 Binding Motifs

We then investigated our library of fragments with DiLiMOT [46] to identify cryptic pVHL30 binding motifs. According to the significance threshold, six motifs were marked as good candidates (Table 2). The two most represented are VGxxxK and PxxxVxxN; each shared among four different pVHL30 interactors. Further, both these putative motifs are present in the EEF1A1 that is a well-known pVHL30 binder [47]. This evidence supports their possible involvement in pVHL30 binding. The remaining proposed motifs are rich in lysines (i.e., GxxKxxK, KKKxK, KxKxKxK, KxxxPK). Lysine is a positively charged amino acid as well as it is a target site of post-translational modifications. According to their electrostatic net charge, these motifs could likely mediate binding with the pVHL30 N-terminal tail, which is characterized by a strong concentration of negatively charged residues [8]. The possibility to turn off the pVHL30 interaction with these motifs by lysine methylation is fascinating. It should be mentioned that these last four motifs were predicted with a *p*-value below the significance threshold; however, this does not completely exclude their reliability, as real binder motifs can still occur also with a less significant value. 

### 3.5. Mutations Found in Cancer Affect the pVHL30 Binding Motifs

We decided then to verify whether cancer-associated mutations localize within the pVHL binding fragments. A search against COSMIC [48] identified a number of mutations found in tumors (i.e. adenocarcinoma, squamous cell carcinoma, malignant melanoma and clear cell renal cell carcinoma) localizing within fragments corresponding to SPATA22, ZBTB17, CCT7, MAP1S and PDIA3 proteins. In particular, missense mutations such as the p.Arg89Ile in SPATA22, p.Arg562Cys and p.Arg625Trp in ZBTB17, p.Pro235Ser and p.Glu316Lys in CCT7, p.Arg863Gln and p.Gly891Ser in MAP1S as well as synonymous substitutions pSer497Ser in ZBTB17 and p.Leu361Leu in PDIA3 seem to correlate with pathogenic conditions (Table 3). Considering the aforementioned findings, it can be speculated that mutations in these positions can alter the pVHL30 binding affinity, thus conferring pro-oncogenic features to cells harboring them. Further investigations are, however, required to address this evidence.

### 3.6. Pathway Analysis of pVHL30 Binding Interactors

**Figure 3:**
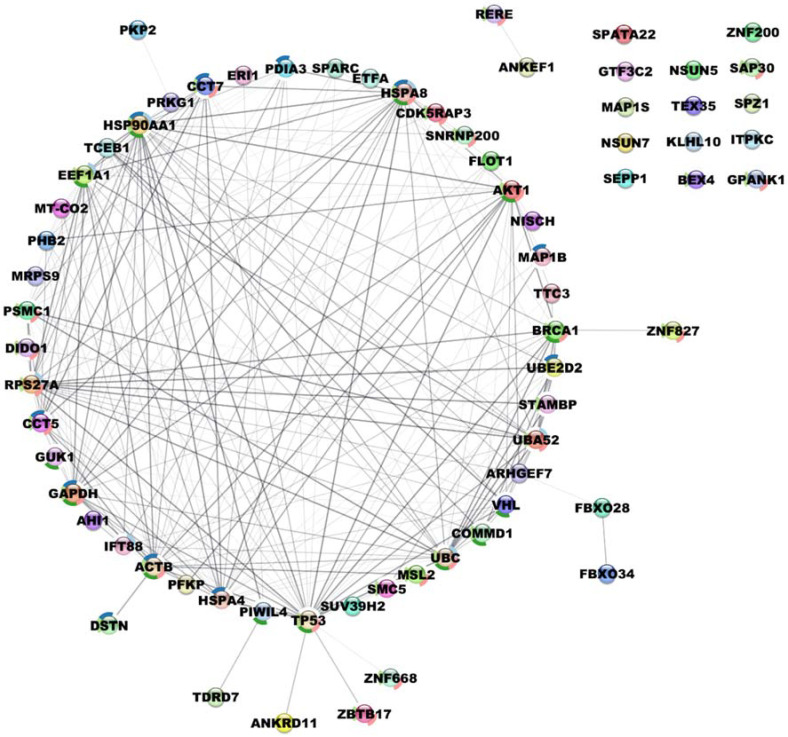
Protein–protein interaction network of novel testis-derived pVHL30 interactors obtained with STRING [32] data. The network is presented as a circular layout, with bubbles representing pVHL30 interactors while edges are functional connections. Intensity in edge coloring reflects the amount of different evidence connecting nodes. A bubble is rounded by a colored circle when the node is shared among different pathways.

To further investigate the pathways in which these novel interactors are involved, a protein interacting network centered around pVHL30 was created using STRING [32]. The resulting network shows no trivial interaction among the 61 proteins except for a few entries which form two small clusters (Supplementary Figure S2). This preliminary network appeared to not have significantly more interactions than statistically expected, thus indicating that our dataset might be either a random collection of proteins not biologically connected, or these pVHL30 interactors have not been extensively investigated and described in STRING. To address this open question, the preliminary interacting network was enriched by including a second layer of interactors and setting “no more than 50 proteins” for each interactor (Figure 3). 

**Figure 4:**
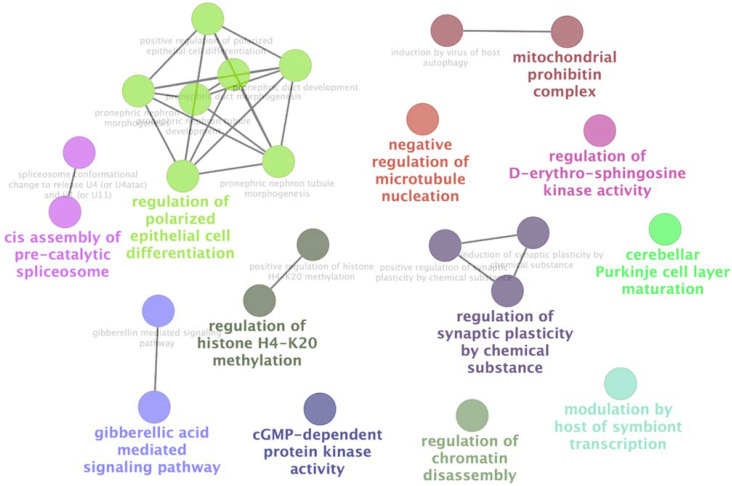
ClueGo [36] representation of gene ontology [30] terms associated with clusters of pVHL30 interactors. Different biological functions are presented as colored bubbles when statistically significant (*p* < 0.05), while light gray text is used for recurrent pathways falling below the threshold value.

By extending the network, a major central cluster composed of 43 proteins highly interconnected was identified. Among them, PSMC1, GUK1 and STAMBP1, EEF1A1, CCT5 and CCT7 are pVHL30 partners identified by the Y2H screening. This cluster seems to have a bi-modal distribution with a most populated fraction containing HIF-1α and several proteins involved in protein degradation. This finding is expected as it is confirmatory of the known pVHL30 involvement in this process. This portion of the cluster also includes PSMC1 and multiple proteins forming the proteasome subunits. The proteasome plays a key role in the maintenance of protein homeostasis by removing misfolded or damaged proteins, which could impair cellular functions, and by removing proteins whose functions are no longer required [49]. It participates in numerous cellular processes, including cell cycle progression, apoptosis or DNA damage repair, processes in which pVHL30 is also involved. On the other hand, in the same cluster were also found several proteins belonging to the chaperonin family (e.g., CCT5, CCT7) known to play a key role in the correct folding of cytoskeleton proteins. This evidence can be related to the pVHL30 involvement in the regulation of microtubule dynamics [50], an example of HIF-1α independent pVHL function. A second interesting cluster includes DIDO1, GTF3C2, SNRNP200 and PFKP. This cluster is composed of 11 nodes and centered around CDC5L, a protein with a key role in cell-cycle regulation and spliceosome activation [51]. Pathway analysis showed that clusters formed by the novel pVHL30 interactors are enriched in multiple biological processes such as regulation of epithelial cell differentiation, synaptic plasticity, histone methylation, chromatin disassembly and negative regulation of microtubule nucleation (Figure 4).

Collectively, these findings highlight already known pVHL30 functions. Moreover, a number of interactors identified in our screening showed no direct interconnections, suggesting that pVHL30 may directly link these proteins, therefore putatively also exerting novel not yet characterized functions.

### 3.7. pVHL30 Interaction with 3’- or 5’-Untraslated Regions (UTR)

In our library screening we also identified another 86 entries that correspond to peptides containing a non-coding region, i.e., 3’- or 5’-untraslated regions (UTR). Together, the Y2H library screening produced two different datasets, one accounting for 142 entries corresponding to 61 different proteins and a second dataset collecting another 86 pVHL30 interacting amino acid sequences not corresponding to individual coding regions. It is known in the literature that Y2H screening results in identification of many out-of-frame (OOF) clones that encode peptides with no sequence homology to known proteins [52]. These OOF clones typically generate during library construction from cloning restriction-digested cDNAs fused to the AD. Nevertheless, deeper analysis of these peptides could still reveal common short linear motifs (SLiMs) responsible for their selection. In other words, they can represent a further reservoir of putative new linear motifs able to mediate association with pVHL30 (Supplementary Table S3).

## 4. Discussion

Y2H screening is a useful technique vastly used to construct extensive protein–protein interaction maps for humans and several model organisms [53]. In this study, we adopted this approach to investigate the interactome around the human pVHL30 in testis. The analysis identified 260 positive clones that were further classified into two main groups: 61 human proteins and 86 peptides derived from noncoding regions. We found six known pVHL30 interactors that were used to confirm the screening system reliability. Indeed, pVHL30 was correctly detected as binder of Elongin C, EEF1A1, CCT5, CCT7, SNRNP200 and UBE2D2 interactors, suggesting the general ability of this technique to find out in vivo real associations outside the physiological context in which they normally occur. On the other hand, this approach allowed us to also identify 55 novel pVHL30 interactors, including proteins involved in relevant cellular pathways, such as the cell-cycle regulation, DNA damage repair, apoptosis and cytoskeleton regulation. These novel interactors were preliminary investigated by searching their connections at the biochemical level. We observed that pVHL30 seems to act as a hub protein connecting all the new 55 binding partners, whereas no trivial interconnections were highlighted among them. To interpret this evidence, it could be argued that pVHL30 may be responsible for their specific degradation in testis tissue, thus indicating that this tumor suppressor may have a relevant yet under-investigated role in this organ. Interestingly, in multiple cases our analysis suggested specific pVHL30 binding motifs which represent a valuable starting point for the identification of functional linear motifs. As expected, our screening highlighted novel tissue-specific interaction of pVHL30 with proteins relevant for the correct testis function. Among them, we found proteins regulating spermatogenesis, such as the Piwi-like protein 4 (PIWIL4), spermatogenic leucine zipper protein 1 (SPZ1) and spermatogenesis-associated protein 22 (SPATA22), indicating that pVHL30 could be functionally linked to sperm cell proliferation and differentiation also in humans. The PIWIL4 is known to represses transposable elements and prevents their mobilization during spermatogenesis, ensuring germline integrity [54]. This protein also plays crucial roles in somatic cells, such as participating in the correct maintenance of the retinal epithelial through the Akt/GSK3alpha/beta signaling axis [55] and regulating pancreatic beta cell function [56]. Both these tissues are target sites for cancer insurgence in VHL syndrome, whose affected patients develop retinal hemangioblastomas and pancreatic cystadenomas. A possible role for the interaction between pVHL30 and PIWIL4 in the formation of these two cancers is at least fascinating and will require further investigation. We also found interactions with multiple proteins regulating metabolism, gene expression and cell-cycle progression. These findings further support an implication of pVHL30 in sperm cell maturation. In this sense, its association with Prohibitin-2 is of interest. In mitochondria, Prohibitin-2 is a chaperone-like acting protein involved in the stabilization of mitochondrial respiratory enzymes [57], a role which is critical for sperm cell development. Indeed, to allow sperm survival during its scrotal storage, the balance between oxidative metabolism and redox homeostasis has to be finely tuned and sophisticated regulatory mechanisms have been evolved by nature [58]. Considered the main pVHL30 function in mediating the hypoxia response, the interaction with Prohibitin-2 opens possible new biological roles for this protein. As an example, epididymal cystadenoma (Ecs) is frequently found in association with VHL disease; however, only little is known about the specific pVHL30 functions in this tissue. Our findings could help to shed new light on this type of cancer. Similarly, we also found interaction with CTSD, a lysosomal aspartyl protease proposed as a relevant diagnostic and prognostic biomarker for RCC [59]. Indeed, increased excretion of CTSD in RCC is not a consequence of its increased transcription [59]; rather, it is thought to be due to protein relocation or other mechanisms. The biological meaning behind CTSD association with pVHL30 is currently unknown; however, based on our findings we speculate that pVHL30 may be involved in the degradation of CTSD as the VHL gene is lost or nonfunctional in over 70% of sporadic RCC [60,61]. Another interesting point to discuss is the ability of pVHL30 to recognize and interact with the UTR regions, as shown by the interaction with 86 entries codifying for out-of-frame clones. The UTR regions are known to have key roles in post-transcriptional gene regulation for the maintenance of cellular homeostasis as they contain different regulatory elements involved in pre-mRNA processing, mRNA stability and translation initiation [62]. In a contest of cancer-induced genetic instability, it can be proposed that these UTRs may fuse with regular codifying regions yielding chimeric protein products able to associate pVHL30. Thus, it can be of interest to investigate whether this specific gene rearrangement happens in cancer cells and how it impacts pVHL30 functions.

Collectively, our library screening showed that pVHL30 can interact with multiple new proteins, suggesting novel testis-specific functions. Further investigations for each new pVHL30 interactor will be necessary to understand the functional meaning beyond these binary associations.

## 5. Conclusions

We described the testis-specific proteome around the human pVHL30 obtained by library screening. Our approach identified 55 novel pVHL30 interactors, with multiple proteins directly involved in spermatogenesis, reproductive metabolism and cancer. While further study is warranted to elucidate the exact role of these new interactions, we demonstrated that pVHL30 can bind tissue-specific interactors and suggested novel roles for this oncosuppressor protein. 

## Figures and Tables

**Table 1:** List of proteins interacting with pVHL30. Known pVHL30 interactors are presented in grey.

Protein Name	UniProt ID	Function	n° Hits
Elongation factor 1-alpha 1 (EEF1A1)	P68104	protein biosynthesis	17
Elongin C (ELOC)	Q15369	protein degradation	6
T-complex protein 1 subunit eta (CCT7)	Q99832	actin/tubulin folding	4
Ubiquitin-coniugating enzyme E2 D2 (UBE2D2)	P62837	protein ubiquitination	2
T-complex protein 1 subunit epsilon (CCT5)	P48643	actin/tubulin folding	1
U5 small nuclear ribonucleoprotein 200 kDa helicase (SNRNP200)	O75643	RNA splicing	1
Putative methyltransferase (NSU7)	Q8NE18	methylation	12
Spermatogenesis-associated protein 22 (SPATA22)	Q8NHS9	germ cell division	10
Zinc finger and BTB domain-containing protein 17 (ZBTB17)	Q13105	cell cycle regulator	9
Microtubule-associated protein 1S (MAP1S)	Q66K74	apoptosis	4
Protein disulfide-isomerase A3 (PDIA3)	P30101	protein folding	4
Death-inducer obliterator 1 (DIDO1)	Q9BTC0	tumor suppressor	3
Electron transfer flavoprotein subunit alpha, mitochondrial (ETFA)	P13804	electron transport	3
26S proteasome regulatory subunit 4 (PSMC1)	P62191	protein degradation	3
Histone deacetylase complex subunit (SAP30)	O75446	deacetylation	3
E3 ubiquitin-protein ligase (TTC3)	P53804	ubiquitination/protein degradation	3
Ankyrin repeat and EF-hand domain-containing protein 1 (ANKEF1)	Q9NU02	n.d.	2
Ankyrin repeat domain-containing protein 11 (ANKRD11)	Q6UB99	chromatin regulator	2
Guanylate kinase (GUK1)	B1ANH3	phosphorylation	2
Protein BEX4 (BEX4)	Q9NWD9	microtubule deacetylation	2
Cytochrome c oxidase subunit 2 (MT-CO2)	P00403	oxygen reduction	2
Prohibitin-2 (PHB2)	Q99623	transcription inhibitor	2
Piwi-like protein 4 (PIWIL4)	Q7Z3Z4	tumor enhancer	2
cGMP-dependent protein kinase 1 (PRKG1)	Q13976	protein phosphorylation	2
Arginine-glutamic acid dipeptide repeats protein (RERE)	Q9P2R6	cell survival control	2
Structural maintenance of chromosomes protein 5 (SMC5)	Q8IY18	DNA repair	2
STAM-binding protein (STAMBP)	O95630	protein degradation	2
Testis-expressed protein 35 (TEX35)	Q5T0J7	n.d.	2
Jouberin (AHI1)	Q8N157	ciliogenesis	1
Rho guanine nucleotide exchange factor 7 (ARHGEF7)	Q14155	apoptosis	1
Protein BEX2 (BEX2)	Q9BXY8-2	cell cycle regulator	1
Breast cancer type 1 susceptibility protein (BRCA1)	P38398	E3-ub lig/DNA repair	1
CDK5 regulatory subunit-associated protein 3 (CDK5RAP3)	J3QRX0	n.d.	1
COMM domain-containing protein 1 (COMMD1)	Q8N668	protein ubiquitination regulator	1
Copine-5 (CPNE5)	A0A0J9YWA1	dendrite formation	1
Destrin (DSTN)	P60981	actin depolymerization	1
3’-5’ exoribonuclease 1 (ERI1)	Q8IV48	RNA exonuclease	1
F-box only protein 28 (FBXO28)	Q9NVF7	ubiquitination/protein degradation	1
F-box only protein 34 (FBXO34)	Q9NWN3	SRP of E3-ub complex	1
Flotillin-1 (FLOT1)	O75955	caveolae formation	1
G patch domain and ankyrin repeat-containing protein 1 (GPANK1)	O95872	n.d.	1
General transcription factor 3C polypeptide 2 (GTF3C2)	Q8WUA4	DNA transcription	1
Intraflagellar transport protein 88 homolog (IFT88)	Q13099	ciliogenesis	1
Inositol-trisphosphate 3-kinase (ITPKC)	Q96DU7	phosphorylation	1
Kelch-like protein 10 (KLHL10)	Q6JEL2	ubiquitination/protein degradation	1
Microtubule-associated protein 1B (MAP1B)	P46821	microtubule stabilization	1
28S ribosomal protein S9, mitochondrial (MRPS9)	P82933	n.d.	1
E3 ubiquitin-protein ligase MSL2 (MSL2)	Q9HCI7	ubiquitination/protein degradation	1
Nischarin (NISCH)	Q9Y2I1	cell survival/migration	1
Probable 28S rRNA (cytosine-C(5))-methyltransferase (NSUN5)	Q96P11	methylation	1
ATP-dependent 6-phosphofructokinase, platelet type (PFKP)	Q01813	glycolysis	1
Plakophilin-2 (PKP2)	Q99959	cell-cell adhesion	1
Selenoprotein P (SELENOP)	P49908	selenium transport	1
SPARC	P09486	cell growth	1
Spermatogenic leucine zipper protein 1 (SPZ1)	Q9BXG8	germ cell proliferation and differentiation	1
Histone-lysine N-methyltransferase (SUV39H2)	Q9H5I1	chromatin regulator	1
Tudor domain-containing protein 7 (TDRD7)	Q8NHU6	post-transcription regulator	1
Zinc finger protein 200 (ZNF200)	P98182	spermatogenesis	1
Zinc finger protein 668 (ZNF668)	Q96K58	transcription regulator	1
Zinc finger protein 827 (ZNF827)	Q17R98	transcription regulator	1

The grey lines correspond to already known pVHL30 interactors.

**Table 2:** Putative pVHL30 interacting motifs identified by DiLiMOT.

Motif	Scons	N° Protein	*p* Value
VGxxxK	2.22 × 10^−29^	4	2.84 × 10^−5^
PxxxVxxN	3.12 × 10^−24^	4	2.20 × 10^−5^
GxKxxK	1.42 × 10^−22^	4	2.48 × 10^−4^
KKKxK	1.13 × 10^−20^	4	9.00 × 10^−6^
KxKxKxK	1.39 × 10^−18^	4	3.81 × 10^−6^
KxxxPK	3.10 ×10^−18^	5	1.29 × 10^−5^
KxxKxxxP	1.20 ×10^−17^	4	2.37 × 10^−4^
KNxxxK	1.63 × 10^−16^	4	3.15 × 10^−4^
AxxVP	3.45 × 10^−16^	4	2.18 × 10^−4^
KKK	4.05 × 10^−16^	5	2.76 × 10^−4^

**Table 3:** New pVHL interactors found mutated in cancers and related amino acid variants.

Protein	Variants	Pathologic Condition
SPATA 22	p.Arg89Ile	glioma and adenocarcinoma (large intestine)
ZBTB17	p.Arg562Cys	carcinoma (endometrium, thyroid)
	p.Arg625Trp	carcinoma (large cell)
	p.Ser497 =	adenoma (large intestine) and carcinoma (upper aerodigestive tract)
CCT7	p.Pro235Ser	malignant melanoma
	p.Glu316Lys	adenocarcinoma (lung, urinary tract)
MAP1S	p.Arg863Asn	adenocarcinoma (large intestine)
	p.Gly891Ser	carcinoma (urinary tract)
PDIA3	p.Leu361 =	ccRCC (Kidney)

## Data Availability

The data that support the findings of this study are available upon reasonable request.

## Supplementary Materials

The following supporting information can be downloaded at: https://www.mdpi.com/article/10.3390/cancers14041009/s1, Figure S1: Western blot to confirm the expression of pVHL30 as bait in Y2H, Figure S2: Single layer protein-protein interaction network generated with RING database, Table S1: List of proteins interacting with pVHL30 identified testis library screening, Table S2: Regions shared among different clones of a same protein, Table S3: Amino acid sequences of the pseudo pVHL-binding fragments corresponding to 3’- or 5’-untraslated regions.
