# Supplementary material for: Characterization of the pVHL Interactome in Human Testis Using High-Throughput Library Screening
Source: Cancers (Basel). 2022 Feb 17;14(4):1009. doi: 10.3390/cancers14041009 (PMC8869832; doi:10.3390/cancers14041009)
Supplement: Supplementary file 1 [file cancers-14-01009-s001.zip › cancers-1526744-supplementary.pdf]

Supplementary Materials

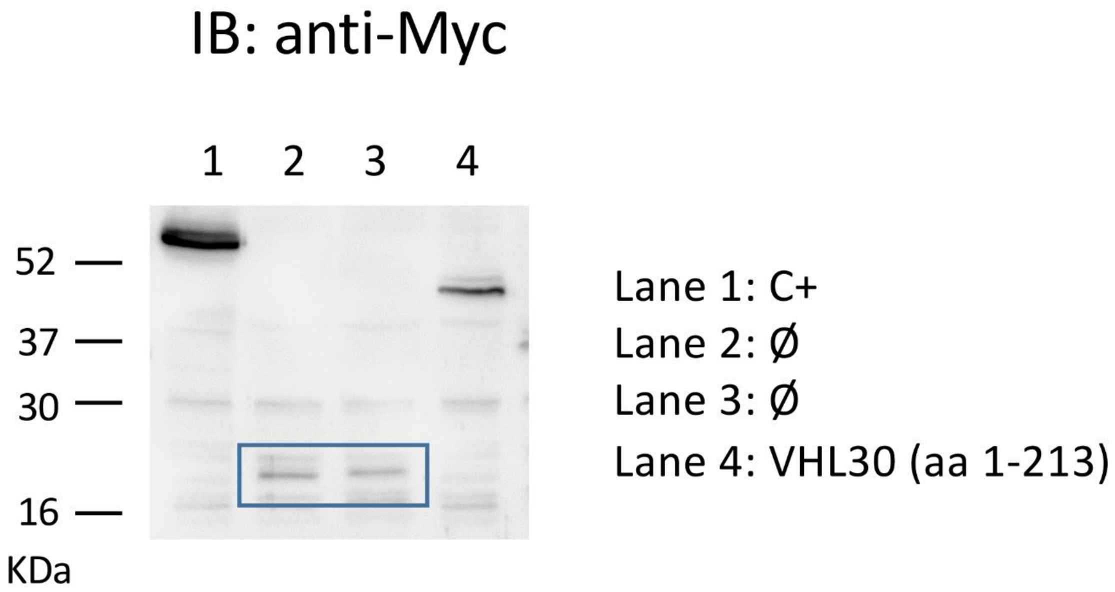

Figure S1. **Western blot to confirm the expression of pVHL30 as bait in Y2H**. Proteins were extracted as described in materials and methods section and visualized in Western Blot. The bait protein (i.e. pVHL30) is detected by anti-Myc (line 4). C+ and Ø correspond to positive (p53-Gal4BD) and negative control (Gal4-BD) respectively.

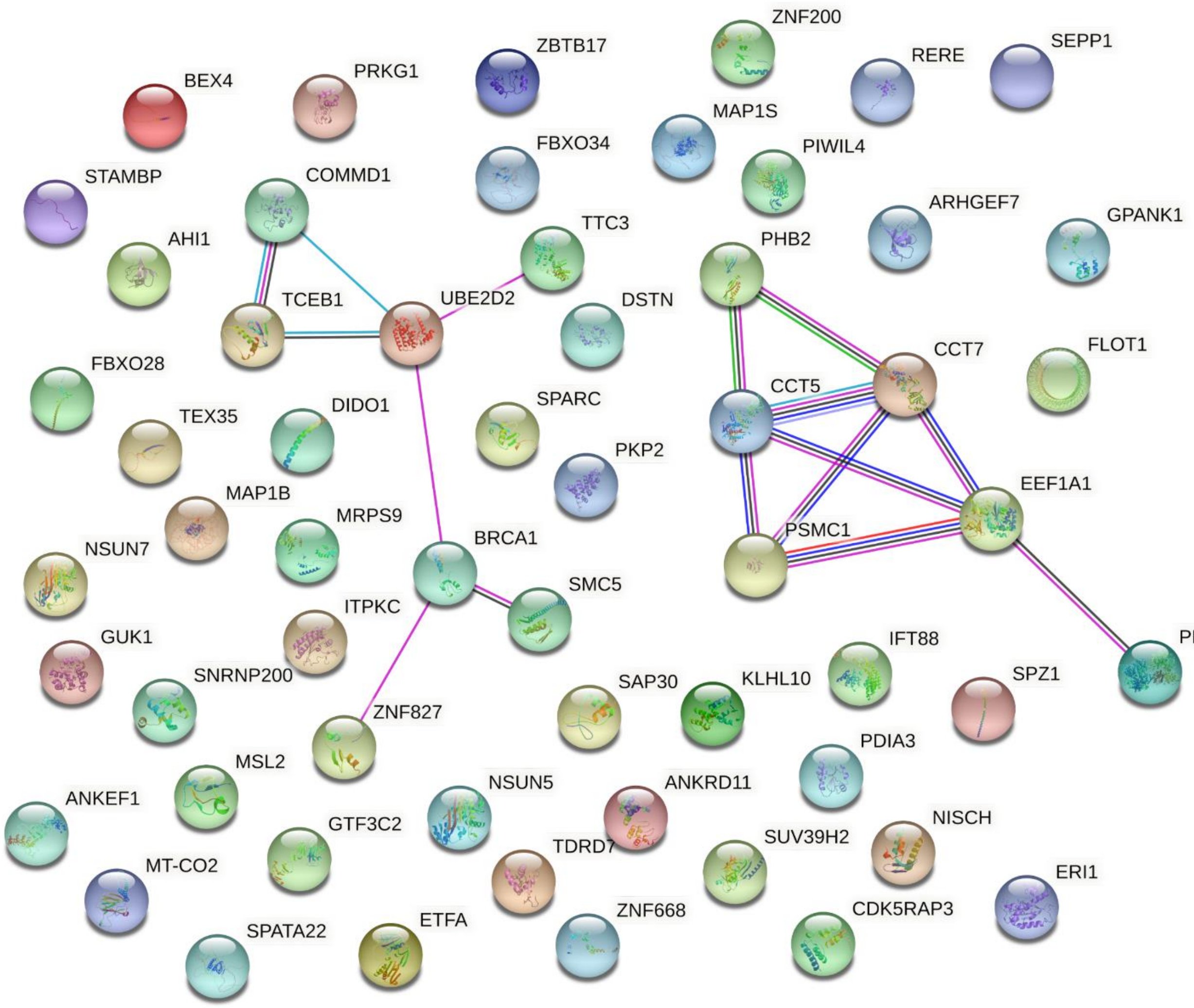

Figure S2. Single layer protein-protein interaction network generated with RING database. Proteins included in the network are presented with colored bubbles while edges are evidence of interactions reported in the literature. Edge coloring reflects different techniques, with green and blue showing in silico predictions while purple and pale blue are for experimentally validated interactions. Finally, black is used for proteins sharing similar co-expression levels.

Table S1. List of proteins interacting with pVHL30 identified testis library screening.

clone	protein name	protein size	binding fragment	function
cl 535	Jouberin	1196	978-1136	ciliogenesis
cl 338	Ankyrin repeat and EF-hand domain-containing protein 1	776	326-624	n.d.
cl 784	Ankyrin repeat and EF-hand domain-containing protein 1	776	325-624	n.d.
cl121	Ankyrin repeat domain-containing protein 11	2663	26-249	Chromatin regulator
cl 237	Ankyrin repeat domain-containing protein 11	2663	26-249	chromatin regulator
cl 491	Rho guanine nucleotide exchange factor 7	782	50-140	GEF apoptosis

cl 5	Protein BEX2	160	6-40	cell cycle regulator
cl 252	Protein BEX4	120	1-120	microtubule Dacetil.
cl 135	Protein BEX4	120	1-120	microtubule Dacetil.
cl 812	Breast cancer type 1 susceptibility protein	1863	1740-1863	E3-ub lig/DNA repair
cl 582	T-complex protein 1 subunit epsilon	541	1-230	actin/tubulin folding
cl 64	T-complex protein 1 subunit eta	543	145-368	actin/tubulin folding
cl 341	T-complex protein 1 subunit eta	543	146-474	actin/tubulin folding
cl 373	T-complex protein 1 subunit eta	543	203-536	actin/tubulin folding
cl 852	T-complex protein 1 subunit eta	543	145-472	actin/tubulin folding
cl 854	CDK5 regulatory subunit-associated protein 3	110	4-110	n.d.
cl 353	COMM domain-containing protein 1	190	1-190	protein ub regulator
cl 119	Copine-5	290	11-239	n.d.
cl 881	Cathepsin D (CTSD)	412	291-412	protease/prot degrad
cl 82	Death-inducer obliterator 1	2220	363-529	tumor suppressor
cl 95	Death-inducer obliterator 1	2220	363-529	tumor suppressor
cl 201	Death-inducer obliterator 1	2220	364-529	tumor suppressor
cl 131	Destrin	165	49-165	actin depolimerization
cl 75	Elongation factor 1-alpha 1	462	233-420	protein biosynthesis
cl 219	Elongation factor 1-alpha 1	462	295-462	protein biosynthesis
cl 175	Elongation factor 1-alpha 1	462	221-462	protein biosynthesis
cl 185	Elongation factor 1-alpha 1	462	295-462	protein biosynthesis
cl 239	Elongation factor 1-alpha 1	462	249-462	protein biosynthesis
cl 308	Elongation factor 1-alpha 1	462	297-462	protein biosynthesis
cl 332	Elongation factor 1-alpha 1	462	297-462	protein biosynthesis
cl 372	Elongation factor 1-alpha 1	462	213-462	protein biosynthesis
cl 900	Elongation factor 1-alpha 1	462	215-324	protein biosynthesis
cl 574	Elongation factor 1-alpha 1	462	165-434	protein biosynthesis
cl 138	Elongation factor 1-alpha 1	462	220-462	protein biosynthesis
cl 154	Elongation factor 1-alpha 1	462	197-434	protein biosynthesis
cl 476	Elongation factor 1-alpha 1	462	297-462	protein biosynthesis
cl 424	Elongation factor 1-alpha 1	462	231-462	protein biosynthesis
cl 324	Elongation factor 1-alpha 1	462	249-462	protein biosynthesis
cl 295	Elongation factor 1-alpha 1	462	288-462	protein biosynthesis
cl 655	Elongation factor 1-alpha 1	462	197-434	protein biosynthesis
cl 39	Elongin-C	112	1-112	protein degradation
cl 590	Elongin-C	112	1-112	protein degradation
cl 216	Elongin-C	112	1-112	protein degradation
cl 217	Elongin-C	112	1-112	protein degradation
cl 454	Elongin-C	112	1-112	protein degradation
cl 936	Elongin-C	112	1-112	protein degradation
cl 450	3'-5' exoribonuclease 1	349	35-209	histone mRNA degrad
cl 633	Electron transfer flavoprotein subunit alpha, mitochondrial	333	201-333	electron transport
cl 799	Electron transfer flavoprotein subunit alpha, mitochondrial	333	203-333	electron transport

cl 89	Electron transfer flavoprotein subunit alpha, mitochondrial	333	200-333	electron transport
cl 312	F-box only protein 28	368	1-126	ub/ prot degradation
cl 807	F-box only protein 34	711	1-53	SRP of E3-ub complex
cl 388	Flotillin-1	427	120-249	caveolae formation
cl 701	G patch domain and ankyrin repeat-containing protein 1	356	1-154	n.d.
cl 251	General transcription factor 3C polypeptide 2	911	1-290	DNA transcription
cl 348	Guanylate kinase (Fragment)	272	72-203	phosphorylation
cl 205	Guanylate kinase	197	21-197	phosphorylation
cl 313	Intraflagellar transport protein 88 homolog	833	39-282	ciliogenesis
cl 286	Inositol-trisphosphate 3-kinase	683	495-617	phosphorylation
cl 736	Kelch-like protein 10	608	472-608	ub/ prot degradation
cl 144	Microtubule-associated protein 1B	2468	2168-2468	microtub stabilization
cl 194	Microtubule-associated protein 1S	1059	778-1053	apoptosis
cl 287	Microtubule-associated protein 1S	1059	863-1059	apoptosis
cl 473	Microtubule-associated protein 1S	1059	677-932	apoptosis
cl 156	Microtubule-associated protein 1S	1059	815-1059	apoptosis
cl 630	28S ribosomal protein S9, mitochondrial	396	45-336	n.d.
cl 309	E3 ubiquitin-protein ligase MSL2	577	375-577	ub/ prot degradation
cl 102	Cytochrome c oxidase subunit 2	227	169-227	oxygen reduction
cl 235	Cytochrome c oxidase subunit 2	227	169-227	oxygen reduction
cl 561	Nischarin	1504	1386-1504	cell survival/migration
cl 236	Probable 28S rRNA (cytosine-C(5))-methyltransferase	429	291-428	methylation
cl 37	Putative methyltransferase NSUN7	718	510-696	methylation
cl 46	Putative methyltransferase NSUN7	718	510-523	methylation
cl 61	Putative methyltransferase NSUN7	718	510-718	methylation
cl 111	Putative methyltransferase NSUN7	718	510-718	methylation
cl 145	Putative methyltransferase NSUN7	718	510-701	methylation
cl 148	Putative methyltransferase NSUN7	718	510-687	methylation
cl 150	Putative methyltransferase NSUN7	718	510-718	methylation
cl 207	Putative methyltransferase NSUN7	718	510-630	methylation
cl 247	Putative methyltransferase NSUN7	718	510-718	methylation
cl 155	Putative methyltransferase NSUN7	718	510-718	methylation
cl 57	Putative methyltransferase NSUN7	718	510-718	methylation
cl 151	Putative methyltransferase NSUN7	718	510-718	methylation
cl 81	Protein disulfide-isomerase A3	505	343-500	protein folding
cl 124	Protein disulfide-isomerase A3	505	345-500	protein folding
cl 187	Protein disulfide-isomerase A3	505	345-500	protein folding
cl 198	Protein disulfide-isomerase A3	505	343-500	protein folding
cl 732	ATP-dependent 6-phosphofructokinase, platelet type	784	562-616	glycolysis
cl 381	Prohibitin-2	299	42-299	transcription inhibitor
cl 439	Prohibitin-2	299	42-290	transcription inhibitor
cl 218	Piwi-like protein 4	852	776-852	tumor enhancer
cl 815	Piwi-like protein 4	852	777-852	tumor enhancer

cl 600	Plakophilin-2	504	472-504	cell-cell adhesion
cl 104	cGMP-dependent protein kinase 1	376	320-376	phosphorylation
cl 226	cGMP-dependent protein kinase 1	376	320-376	phosphorylation
cl 261	26S proteasome regulatory subunit 4	440	1-250	protein degradation
cl 318	26S proteasome regulatory subunit 4	440	1-294	protein degradation
cl 387	26S proteasome regulatory subunit 4	440	1-180	protein degradation
cl 911	Arginine-glutamic acid dipeptide repeats protein	1566	59-134	cell survival control
cl 378	Arginine-glutamic acid dipeptide repeats protein	1566	59-134	cell survival control
cl 182	Histone deacetylase complex subunit SAP30	220	61-210	deacetylation
cl 892	Histone deacetylase complex subunit SAP30	220	77-220	deacetylation
cl 637	Histone deacetylase complex subunit SAP30	220	64-220	deacetylation
cl 475	Selenoprotein P	382	119-299	selenium transport
cl 256	Structural maintenance of chromosomes protein 5	1101	1-113	DNA repair
cl 400	Structural maintenance of chromosomes protein 5	1101	1-114	DNA repair
cl 68	U5 small nuclear ribonucleoprotein 200 kDa helicase	2136	1229-1334	RNA splicing
cl 72	SPARC	303	133-303	cell growth
cl 96	Spermatogenesis-associated protein 22	363	1-249	germ cell division
cl 100	Spermatogenesis-associated protein 22	363	27-269	germ cell division
cl 137	Spermatogenesis-associated protein 22	363	58-324	germ cell division
cl 215	Spermatogenesis-associated protein 22	363	22-189	germ cell division
cl 231	Spermatogenesis-associated protein 22	363	20-326	germ cell division
cl 830	Spermatogenesis-associated protein 22	363	22-286	germ cell division
cl 97	Spermatogenesis-associated protein 22	363	62-363	germ cell division
cl 158	Spermatogenesis-associated protein 22	363	20-356	germ cell division
cl 915	Spermatogenesis-associated protein 22	363	20-115	germ cell division
cl 393	Spermatogenesis-associated protein 22	363	24-294	germ cell division
cl 58	Spermatogenic leucine zipper protein 1	430	300-430	germ cell prolif and diff
cl 642	STAM-binding protein	424	151-424	protein degradation
cl 523	STAM-binding protein	424	151-370	protein degradation
cl 383	Histone-lysine N-methyltransferase SUV39H2	410	293-410	chromatin regulator
cl 880	Tudor domain-containing protein 7	1098	749-913	post-transcr regulator
cl 65	Testis-expressed protein 35	233	1-170	n.d.
cl 418	Testis-expressed protein 35	233	1-196	n.d.
cl 165	E3 ubiquitin-protein ligase TTC3	2025	998-1185	ub/ prot degradation
cl 166	E3 ubiquitin-protein ligase TTC3	2025	998-1185	ub/ prot degradation
cl 249	E3 ubiquitin-protein ligase TTC3	2025	998-1247	ub/ prot degradation
cl 691	Ubiquitin-conjugating enzyme E2 D2	147	8-147	protein ubiquitination
cl 533	Ubiquitin-conjugating enzyme E2 D2	147	8-112	protein ubiquitination
cl 48	Zinc finger and BTB domain-containing protein 17	803	459-662	cell cycle regulator

cl 56	Zinc finger and BTB domain-containing protein 17	803	306-450	cell cycle regulator
cl 74	Zinc finger and BTB domain-containing protein 17	803	459-723	cell cycle regulator
cl 524	Zinc finger and BTB domain-containing protein 17	803	459-711	cell cycle regulator
cl 78	Zinc finger and BTB domain-containing protein 17	803	459-711	cell cycle regulator
cl 426	Zinc finger and BTB domain-containing protein 17	803	460-741	cell cycle regulator
cl 757	Zinc finger and BTB domain-containing protein 17	803	461-793	cell cycle regulator
cl 754	Zinc finger and BTB domain-containing protein 17	803	487-757	cell cycle regulator
cl 270	Zinc finger and BTB domain-containing protein 17	803	461-789	cell cycle regulator
cl 141	Zinc finger protein 200	395	200-359	spermatogenesis
cl 22	Zinc finger protein 668	619	319-365	transcription regulator
cl 768	Zinc finger protein 827	1081	1013-1076	transcription regulator

Table S2. Regions shared among different clones of a same protein (* one clone was excluded from the mapping). Numbering refers to canonical sequences deposited in the UniProt database.

Protein	N° hits	Shared region
EEF1A1	17	297-324
NSUN7	12	510-523
SPATA22	10	62-115
ZBTB17	9*	487-662
CCT7	4	203-368
PDIA3	4	345-500
MAP1S	4*	863-1053
DIDO1	3	363-529
PSMC1	3	1-180
SAP30	3	77-210
ETFA	3	203-333
TTC3	3	998-1185

Table S3. Amino acid sequences of the pseudo pVHL-binding fragments corresponding to 3′- or 5′-untraslated regions.

>ID_66HI67_11A_cl20
WPGTGKMPNKKTKEGSRPPRRMKKNPAIWEGVKARSLEEISGGGELGDLSLIFDGPYLIGILSTMKREMM
>ID_66HI70_5B_cl25
WPGGDSAGGCTLQMREQHLMAFSSRGTSQPDPATAAAGGRXNCGXSRRVQPAQKKKKKKKIFGSKTVQTTL
>ID_66HI71_6B_cl28
PGTGKMPNKKTKEGSRPPRRMKKNPAIWEGVKARSLEEISGGGELGDLSLIFDGPYLIGILSTMKREMM

>ID_66HI73_8B_cl31
WPGTGKMPNKKTKEGSRPPRRMKKNPAIWEGVKARSLEEISGGGELGDLSLIFDGPYLIGILSTMKREMM
>ID_66HI74_11B_cl34
PLWRGASXGGFCXKRSSGVXRXHAGNIXWCPCEQXXVLQNPAASEHPEEAISALH
>ID_66HI76_1C_cl36
PGEVAVSRDRAIALQPGQQEGNSVSKKKTKTNRKRKYTSLRRYSEEILFNNKEDLCTFVRLFTVKPQPASPSSVLTIATIRGKEMNLF
>ID_66HI82_10D_cl55
PQAVVSTLSGHYGREIKGYRAELIKLQIQVFSR
>ID_66HI84_1E_cl59
PGTGKMPNKKTKEGSRPPRRMKKNPAIWEGVKARSLEEISGGGELGDLSLIFDGPYLIGILSTMKREMM
>ID_66HI85_1B_cl23
PGAGNGRGSVSPSGRARSAPAPVVVAAQGSCSWAGRPPAGSQRRRPAGGPEAARALREGRPCTDYVGKGLPSFLDISFTSCGVDQGL
TELDIQDGALIYLVVGAGYPLGLQMGLLIRIPHVASPPGESLST
>ID_66HI87_3D_cl50
LWPGRCDLNVGAISHISA
>ID_66HI93_11E_cl67
CAVATPIGAECTEWNKTETCPQEQSRSCIECDVCVINMFHTNKACQLKKFEHLGYYFLWL
>ID_66HI95_1F_cl517
CAVATPIGAECTEWNKTETCPQEQSRSCIECDVCVINMFHTNKACQLKKFEHLGYYFLWL
>ID_66HI97__6F__cl76_
PGSKKKKKKIRETKLAFLANNIIIYDANPREPCWILLVAGHKINIQGLNISTY
>ID_66HJ00__12F__cl92
PGALGDRDCPQEEMVPPPPCPLSCPQLSPHHALSFKGLVRPFPVTVTEAGDGGMGRRRKETPPAYLPGQRPQKPETDLGVEQQQLGE
DPAGVQEPACLAAQPAPTLLEASILCSGEGPLVSSEMASACFLPSVLENTYFLFPPLPSPTWTCSEPCVLWCVASLPSLFSSFWILSYLHPQ
SLPFILPLLYPETAFCPGSHQIDPVLPLVK
>ID_66HJ03__6G__cl98
FWKHVARLRFFYKYGEGKRQGVDLHREVEGRGSAGF
>ID_66HJ06__12G__cl109_
GGDSAGGCTLQMREQHLMAFSSRGTSQPDPATAAAGGRGTAGKPKSPTCSKKKKKKKNLRVKNCSNYSLRAPFGVKTVYLLYGYGDSK
SSDLVTYISQFLVV
>ID_66HJ08__4H__cl116
TLERNHMNAIYVVKPSIEVTTLDFIEEFTLERNHMYVLYVGKPLVNFLTLDNMRELTLKKQ
>ID_66HJ13_Clone_99
GGIQPLLRDKCSDNSSCHPTPWVLTASGSPAISGTNPADPPGRGVCSPGRPAPAAGRTGQESCRTGTQGAGAAEHCSQLACETEQLAP
SALVVPCEALLLSGFLHRDPCRLPADMQDALLSVDVAFSDSVSEPACLPGLVLGQQLQGSGLWPLHPVVSDLHSLCLPLLVPTHL
>ID66HJ15_Clone_112
RGISSDIFILAESSRREGPRSDLAVPEEHGTLYVCPCFSLGVVTLKNILHFGIQF
>ID66HJ16_Clone_136
QGGHAPGPGHGPAGALGTQAAGGHRVHAVWALDATLSDPAGAHGHHLAREARGRTLPGATALCEGFLQTPGLLQQLCDTTSLPLHE
PELPQQAPTADEKAALRGPALLPGPHGGAAGAGVGGPALLGRRDSGGRRALSYPGRSPHPSRRRRAAGREAGGVFFLKFPFSHKCHS
WAKAVVPVAGIWLESPRGLCVSQTRSSGPHPAKASSPSASSAFICNEVMKGLKASIYTL
>ID66HJ18_Clone_139
PGGQRANGPSRREPGDYAAPCRSTFRLGGSTMKKVKKKRASDSRRPAFLLLCHLPLQLRLLASSGAMP
>ID66HJ36_Clone_230
RGAQQRRGSKRKWDASPLVSSTLEKDVVLQVQTFSPTWGVSPHCIPPHLFLPSCTVSFECAALSGLGASLVLQLGHIRHTDHPVLLLPLE
DPEQPLF
>ID66HJ38_Clone_233
PTRPSWRSSRGRKVQPELDSGLQHPGVVPSDHEEPRPAGPTADSAQSMQGTLVQLSWGRQRPGLPKGQRVVERRGRKGSPRARGP
QRTRVVAGPSISADLPGAQSFPALSQAGHERVHFSSAGWAGEPLCAAVWGEPCCGLLVVVPLPTCSPVGKKGRKQVPPGVLLPKPPT
>ID66HJ43_Clone_695
LDNSCPRWSLALSPRLECSGMISAHCKLHLPSSRHSPASASQVAGTTGAHHQARLIFLYF
>ID66HJ44_Clone_246
KEGKPLLNVAKETDCPMLCRKLPHQVKCTSEQRQAWEGGPKATWQTTGQGTRSLLLPKHLDASSMGWCAGLVKADGGAEPTCKIHF
LSIRSLDKSMNLHVRKREDVVLKRNKKTKPTRKENPRVSGLELVLHLERAPGTCP
>ID66HJ49_Clone_265_
GGDSAGGCTLQMREQHLMAFSSRGTSQPDPATAAAGGRGTAGKPKSPTCSKKKKKKKNLRVKNCSNYSLRAPFGVKTVYLLYGYGDSK
SSDLVTYISQFLVV
>ID66HJ50_Clone_267

GAEVMPLHSNLADRARLCLKKRRKEKKTMTRKGVGKEDNFSEPKSEMPTGVFCTCIRLGESKVAAVLEVEKQSFLRSLATALDFEW
>ID66HJ59_Clone_634
PGTGKMPNKKTKEGSRPPRRMKKNPAIWEGVKARSLEEISGGGELGDLSLIFDGPYLIGILSTMKREMM
>ID_66IA08_cl369
GTSAFENFSMPMRRESEEEGAKVSRKKTRGAQNWKAQRSRPMLKQGRVMKVMLVMQPHTPCEGLGLYPLSNESHRKQRDQNFTLE
RLLWGLNGCGKKRVMD
>ID_66IA09_cl340
GTVAHACNPSTLGGQGGQITRSGVQDQPGQQSETPSVLKIQKISQAWWCMPVIPATWEAEAGELLEPGRQRLQPAKITPLHSSLGYRV
CLCLKKKKKSQAYDHVLEQAYKDINGWSLLL
>ID_66IA11_cl940
PGQQQYCFGSNSCLSNCSCETMDRLCLSLLIYERGMLSIYLLESLPLLILCSG
>ID_66IA12_cl346
PGGGGGGSEPRSCYCTLAWVTEGGTVSKPPPKKKLHSTCFIIYLPLYPSFCSSVVLF
>ID_66IA16_cl813_
CSSSAKRQTASSSGFLTPVPPDWETPPSKGQQTPHTGELQLAYGGFPSGMSLPEEGIAVILAVLQPPLVIPRQTGSGVDLKQTPADLQRR
DLTVKRKTNKQKGIVLTSTKRTSTQKPHTNVTNIKDERYINPER
>ID_66IA17_cl822
ECSSSAKRQTASSSGFLTPVPPDWETPPSKGQQTPHTGELQLAYGGFPSGMSLPEEGIAVIFQSFR
>ID_66IA21_cl832
PGAWWRAPVIPALWEAEVGGLLESRSLRPAWATWRDPVSTKRKREKKKAGTVILISSLGYPQEWTFKWL
>ID_66IA22_cl833
RGTTEKGMRGLGAPQPEKRDALLKTRGQQGCFQTRHPEGPGDGAVAFILLIPVNTAPSITLSLSSHSYLHESLGRKSLRRTCKTVKPS
>ID_66IA23_cl848
GGRGTIIYVVSYVFPQWGWAPLLKL
>ID_66IA24_cl849
WPGGAAPSPTTHQCLLPPALAFQVAGITDLCHHAQLIFVFLVETEFRHVGQAGLELLTSGDLPALASQSVGITGMSPTAPGLYVFF
>ID_66IA26_cl943
RGLLSLREVVTPFTATVTAQVLTLQEGGRWGPLGLLVPSWCVAGGPSHVPPGGPHSLADEQIPLATHVLSDISDVLLLCEDQCFLSLHFLP
NLG
>ID_66IA27_cl865
PGTGLASFEIESTSPFLFVMKTPAPAPLGCYLTPVQRKGGKLRRKGRDRQDSISQLVRIRALPTIFSYQKSTEFPRGR
>ID_66IA28_cl944
PGGNCQWLGVITSGCCHGTLMGVPYGKLVLNLVQCPSPTLELSPAFYLNFGDICLWWP
>ID66IA47_cl_883
PGEKDIFRYTGSQLIYLPRAFSQETPPKHVHKPRKRKEKTRDPVILIYYRAEKEFIRTIIL
>ID66IA50_cl_888
GGESVASRSTQGVACCIPTVVLQEVFLVAGDTCCASGEDVQCLQHSLRMAGLTPVIPTLCEAEASRSPEVRSWRPAWSTW
>ID66IA51_cl_897
PGGIQPLLRDKCSDNSSCHPTPWVLTASGSPAISGTNPADPPGRGVCSPGRPAPAAGRTGQESCRAGTQGAGAAEHCSQLACETEQLA
PSALVVPCEALLLSGFLHRDPCRLPADMQDALLSVDVAFSDSVSEPACLPGLVLGQQLQGSGLWPLHPVVSDLHSLCLPLLVPTHL
>ID66IA52_cl_419
PGGIQPLLRDKCSDNSSCHPTPWVLTASGSPAISGTNPADPPGRGVCSPGRPAPAAGRTGQESCRTGTQGAGAAEHCSQLACETEQLA
PSALVVPCEALLLSGFLHRDPCRLPADMQDALLSVDVAFSDSVSEPACLPGLVLGQQLQGSGLWPLHPVVSDLHSLCLPLLVPTHL
>ID66IA54_cl_950
GGALGCPYIFTPWVILVQGLLVEPSKGNCYYRDLLIGDW
>ID07HJ12_cl_87
GGCSEPRSCPCTPAWATERGSISKKKKRKEKKICPLPSEPEEKRKKICSVVLPLRFIFVLL
>ID07HJ18_cl_545
GPGVACKGREQLFSRKDPKGSKAKGAICFPTDSEDKQEKCPVSPILLVLIFQ
>ID07HJ20_cl_564
GDGRKSPVLGQLETVVVTEITLPKESYFANYLLQRRWPMCFVTEPDWFPISQDFAL
>ID07HJ21_cl_572
GGNCQWLGVITSGCCHGTLMGVPYGKLVLNLVQCPSPTLELSPAFYLNFGDICLWWP
>ID07HJ23_cl_127
GCSGHLMYQTQRHESIPRWYPVINHLFWIQSPRRQVESWGKAHRKKAVLKAASRPSLSLAKTHQGIQKVHPQRKRPSSSLKPQAAQTK
AAHTAPQEVWLDHVDRIRSAAPVARGTPAQTTASSLLRALYPFGDEVYFDS
>ID07HJ32_cl_599
PVLAVLRADVSLCSFRETAAISPLAVTWSSTLPPERGCPGVCPGEPQKPATLGEKGQGPEGLAFFLL

>ID_07IA32_cl_464
GALGDRDCPQEEMVPPPPCPLSCPQLSPHHALSFKGLVRPFPVTVTEAGDGGMGRRRKETPPAYLPGQRPQKPETDLGVEQQQLGED
PAGVQEPACLAAQPAPTLLEASILCSGEGPLVSSEMASACFLPSVLENTYFLFPPLPSPTWTCSEPCVLWCVASLPSLFSSFWILSYLHPQSL
PFILPLLYPETAFCPGSHQIDPVLPLVK
>ID_07IA31_cl_459
CRGTQVPGCDRTGHLVSPQSHSDCHWFPIWI
>ID_07IA29_cl_27
GGDSAGGCTLQMREQHLMAFSSRGTSQPDPATAAAGGRGTAGKPKSPTCSKKKKKKKNLRVKNCSNYSLRAPFGVKTVYLLYGYGDSK
SSDLVTYISQFLVV
>ID_07IA28_cl_26
TGKMPNKKTKEGSRPPRRMKKNPAIWEGVKARSLEEISGGGELGDLSLIFDGPYLIGILSTMKREMM
>ID_07IA27_cl_910
GPEAGHSHVAVGRVELLVVNEVDHPGEGLPADATLEGSFGAARGHWALGSIPGWDGLPGALAPGGFLPRWLQQVDLAMLSEDGVV
QEDLATLGARKGPFLAMGLLVLDEVGDPGEDHPTVGTLEGPPTSGPTRHYRDACNSLLGTTFGVLEGWLVAAALVSVPVSPSGTDSLST
WDPWGLEITVPSARRSEEGPAKSHSGSSQILGFRGKRARGPCGEQ
>ID_07IA24_cl_17
GTGKMPNKKTKEGSRPPRRMKKNPAIWEGVKARSLEEISGGGELGDLSLIFDGPYLIGILSTMKREMM
>ID07IA16_cl_427
FAPSLQLAPELCTRGFGDHTGTCLPRFPWLTFLQTLDLPGLGIGGQERGPHPAPSRPVCTVHRGVIRTERGASSMCNLAAAPDIPGLGVL
IYF
>ID07IA10_cl_411
PGGPAGRRSGSRVRGTSGVKSPQAGNKPRPNSHPPAFLQLDLEAASPVWDAADARLLALAASPRLRSSVAAATVTRSPRPDYLEDELFS
SATCYYVIF
>ID07IA09_cl_410
GGDSAGGCTLQMREQHLMAFSSRGTSQPDPATAAAGGRGTAGKPKSPTCSKKKKKKKNLRVKNCSNYSLRAPFGVKTVYLLYGYGDSK
SSDLVTYISQFLVV
>ID07IA07_cl_398
TGKMPNKKTKEGSRPPRRMKKNPAIWEGVKARSLEEISGGGELGDLSLIFDGPYLIGILSTMKREMM
>ID07HJ98_cl_377
LAKIIWGPSLRKGCWCICGCCRPSSLSYQSLLPARAVTTQPVTLRRRVKKPTSPQARRMADCQYHT
>ID07HJ96_cl_365
PGRRERNEEQRDMWRREKAGNKLGIKSQSRECKRKSEVKKVKRKCRVNRVLRNNGLPISEVPIQRVGLFSYVTSLRNNTNTFIKKPELCYI
INSSELRSLPKMPTLPTGAILHHLPNKRI
>ID07HJ95_cl_354
GSSVRGVRPLAPDHHVGFRHQPLRGPSGCKPLPGSLCDPADQRPAGRPGGIQPLLRDKCSDNSSCHPTPWVLTASGSPAISGTNPADP
PGRGVCSPGRPAPAAGRTGQESCRAGTQGAGAAEHCSQLACETEQLAPSALVVPCEALLLSGFLHRDPCRLPADMQDALLSVDVAFSD
SVSEPACLPGLVLGQQLQGSGLWPLHPVVSDLHSLCLPLLVPTHL
>ID07HJ92_cl_330
PGTGKMPNKKTKEGSRPPRRMKKNPAIWEGVKARSLEEISGGGELGDLSLIFDGPYLIGILSTMKREMM
>ID07HJ91_cl_315
PTRPSWRSSRGRKVQPELDSGLQHPGVVPSDHEEPRPAGPTADSAQSMQGTLVQLSWGRQRPGLPKGQRVVERRGRKGSPRARGP
QRTRVVAGPSISADLPGAQSFPALSQAGHERVHFSSAGWAGEPLCAAVWGEPCCGLLVVVPLPTCSPVGKKGRKQVPPGVLLPKPPT
>ID07HJ85_cl_323
GGDSAGGCTLQMREQHLMAFSSRGTSQPDPATAAAGGRGTAGKPKSPTCSKKKKKKKNLRVKNCSNYSLRAPFGVKTVYLLYGYGDSK
SSDLVTYISQFLVV
>ID07HJ84_cl_319
GGDSAGGCTLQMREQHLMAFSSRGTSQPDPATAAAGGRGTAGKPKSPTCSKKKKKKKNLRVKNCSNYSLRAPFGVKTVYLLYGYGDSK
SSDLVTYISQFLVV
>ID07HJ77_cl_729
GAGPHSFPGLGFCTLDGPLQNVVQGRHGLLTGHLEVIGVLVCPRQEHGSKQVPSAHKDGVHFANVHPDAFHHHGASGIPPPLTPQHN
QVVAALRGGQVGAGDDHGVGAHRVQHVHHPQGFLLAVDLPATQ
>ID07HJ75_cl_706
GGRCVVTTAAVAVSEAAVAAALALSGICGCLRVSAVPTLLFADPTPSSDPEPTAGAPGNGGLDGLAPAHQGDLEEQDLYDFLYGGVGRT
APRECRRGAEQKIPEVG
>ID07HJ73_cl_699
GLQSESLSQKKKKTKPKTIKKILMSPITFDLFTANYSIFEIILSF
>ID07HJ58_cl_227
GGEGGGCQSWRAGHRGHGGSTLAAAGGQSRGHQPVS

>ID07HJ55_cl_932
GEAEVVVQAREEAGEEEGARIITKGVNLNSISSMEVISIIILDLDREDITLVEATEPYILLELK
>ID07HJ54_cl_646
PGTGKMPNKKTKEGSRPPRRMKKNPAIWEGVKARSLEEISGGGELGDLSLIFDGPYLIGILSTMKREMM
>ID07HJ52_cl_199
SVFRSFSTKRDIFGSYSLNNCIICSFHDEIAAVEKTGGNSRKREKPTSARRARNCPAWVLAFPAGPVRLSDPAWLLGEGRTVLLVEEYVEFL
>ID07HJ50_cl_626
GERERGVNKGLLPEPEPTRESLCAEGGPQVLLLLHLDPFTPSSNRDLDLPSPPSWILLSVVQAFSGALEFPLTFSPASCICCSH
>ID07HJ49_cl_619
GTGLASFEIESTSPFLFVMKTPAPAPLGCYLTPVQRKGGKLRRKGRDRQDSISQLVRIRALPTIFSYQKSTEFPRGR
>ID07HJ48_cl_602
GRGESEVLQGSPDEHSQKQVGRAGSPCLSRGQHHFSTPCHPRRKSFIQKLGEHAQVQLGKWKIRKPGEENQKLDKRRMNSGNTHPTL
SSVTLQGPCKPSRKEHHSGTPSHTCECGTLLGASNKYRITEWPLCCGFSSLLKGRGHLETEAAWLQPLGRFPTPALRRLVVRAPRKSERS
>ID07HJ44_cl_129
PGTVAHACNPSTLGGQGGQITRSGVQDQPGQQSETPSVLKIQKISQAWWCMPVIPATWEAEAGELLEPGRQRLQPAKITPLHSSLGYR
VCLCLKKKKKSQAYDHVLEQAYKDINGWSLLL
>ID07HJ43_cl_565
PGCGAEAQHGRPGPHLPAAPARLGEPASRTGGRTAQPPARHWTGGPPRADPTGAGRVSGKPGVCESAPSTQCQRGQREPPGLGRYC
RGQGAPPEAGRRGAVSSQAWGHLEGSPQQPGARSPAGGSQHWRPRDGAAGAPVSGLPEGHAEAGGHSGTAQQTSPGTAGHSYG
GGVPWQGTTLVTVPGLALERHPVCAVCLLPPARRSVYLDPSPGSRRKERRPGSRGRDWTLWKRSLASFGPPEECRLPGSRDFRLTRWI
MALKPWKYLLLRTPSGSTRKGPGLPKHTILKPPYPRNQASEGSPLWPPFTEVRTLKTQWKTKGSPQGLCQRLHGPGLKA
>ID07HJ42_cl_552
GCGAEAQHGRPGPHLPAAPARLGEPASRTGGRTAQPPARHWTGGPPRADPTGAGRVSGKPGVCESAPSTQCQRGQREPPGLGRYCR
GQGAPPEAGRRGAVSSQAWGHLEGSPQQPGARSPAGGSQHWRPRDGAAGAPVSGLPEGHAEAGGHSGKFNSMMKIPHQTQKKR
SLIRLTIGRAPPWSTHTTYQITLIWPWRPVNSTKAVVSTQSGHYGRGQNLGIPASRTVRKEFSVVSVPHTMAFCYGSLS
>ID07HJ41_cl_543
GPRATRWGQKTSLTQVPNHHYLPRVQVGGVFVHQVRPPEVGTQHLQLALGPSLVVHDFVFCIQKTHFQLELVTALDGDLVIHQPCRR
QERPGLREGHDLGHTNGKAPQ
>ID07HJ40_cl_529
GGCSEPRSCPCTPAWATERGSISKKKKRKEKKICPLPSEPEEKRKKICSVVLPLRFIFVLL
>ID07HJ39_cl_494
GGELGELGWGEPVRTERRNEKKEQKYYFYVIYILLMPNYL
>ID07HJ37_cl_457
LWPGTGKMPNKKTKEGSRPPRRMKKNPAIWEGVKARSLEEISGGGELGDLSLIFDGPYLIGILSTMKREMM
